# Miswak-Infused Glass Ionomer Cement: A Comparative In Vitro Analysis of Antibacterial Efficacy and Compressive Strength

**DOI:** 10.7759/cureus.53484

**Published:** 2024-02-03

**Authors:** Kamala Devi, Jessy Paulraj, Rajeshkumar Shanmugam, Subhabrata Maiti

**Affiliations:** 1 Pediatric Dentistry, Saveetha Dental College and Hospitals, Saveetha Institute of Medical and Technical Sciences, Chennai, IND; 2 Pharmacology, Saveetha Dental College and Hospitals, Saveetha Institute of Medical and Technical Sciences, Chennai, IND; 3 Prosthodontics, Saveetha Dental College and Hospitals, Saveetha Institute of Medical and Technical Sciences, Chennai, IND

**Keywords:** compressive strength, restorative material, modified gic, antimicrobial, miswak

## Abstract

Background: Glass ionomer cement (GIC) restorations are commonly used in primary dentition, due to their aesthetic appeal, self-adhesive nature, and biocompatibility. However, the material's limited antibacterial activity and inadequate mechanical strength highlight the necessity for modifying the material.

Aim: The study aims to evaluate and compare the antimicrobial potency and compressive strength of GIC-incorporated Miswak extract with that of conventional GIC.

Materials and methods: After obtaining the Miswak extract, a modified GIC was formulated by combining the extract with the conventional GIC powder and liquid components, in three different ratios (Powder: Extract and Liquid), Group I (2:1:1), Group II (3:1:2), Group III (3:2:1), and the Group IV as control, which consist of unmodified/conventional GIC. To evaluate and compare the antibacterial efficacy of the modified and unmodified GIC, standard strains of *Streptococcus mutans* and *Lactobacillus *were utilized. For each group, the minimal inhibitory concentration (MIC) assay was tested. For the evaluation of compressive strength, cylindrical moulds were utilized in compliance with ISO 9917-1:2007 standards and tested using the universal testing machine (Instron, ElectroPuls®, Bangalore, IND). The highest force exerted at the point of specimen fracture was recorded to calculate the compressive strength values in MPa. The data obtained were analyzed using the Statistical Package for the Social Sciences (IBM SPSS Statistics for Windows, IBM Corp., Version 24.0, Armonk, NY) software. The statistical analysis was conducted utilizing repeated measures of analysis of variance (ANOVA) to calculate the mean MIC values and compressive strength, with pairwise comparisons assessed using Tukey's post hoc test.

Results: The results proved that the antimicrobial properties of Miswak containing GIC performed better against *S. mutans *and *Lactobacillus *with a statistically significant difference when compared with group IV (p<0.05), it has been found that an increase in the concentration of extract increased the antimicrobial potency. Significant results were obtained in compressive strength where Group II (41.49±3.6) and Group III (15.23±4.96) proved to be weaker than the control (62.69±2.58), while Group I showed no differences from the control group (p>0.05).

Conclusion: It can be concluded that Group I was found to be better in terms of both antimicrobial properties and compressive strength, where no significant difference in compressive strength was identified when comparing Group I with Group IV. Thus, the overall study depicts that a lesser concentration of extract can be the best option in terms of good antimicrobial properties without altering its strength. Hence, the Miswak containing GIC could be a promising restorative material; further studies should include considering intraoral variables such as masticatory stress, moisture levels and in-vivo tests of this combination.

## Introduction

Glass ionomer cement (GIC) materials have a five-decade history in clinical applications. Over time, these materials have undergone modifications to serve various purposes, including lining, bonding, sealing, repairing, and restoring teeth. Although GIC is recognized for its features like fluoride release, tooth colour matching, and adhesion to dental structures, it has drawbacks including sensitivity to moisture, limited antimicrobial activity, prolonged wear problems, and inadequate strength, which may impede its effectiveness [1]. Hence, in situations such as insufficient remaining tooth structure to support material or the inability to withstand heavy occlusal loads, which affect the prognosis, GIC is strictly not recommended. The past decades have shown that the use of these materials is expanding; however, they may hold specific niches of clinical use. Caries is a complex and ever-changing dental condition that arises from a combination of factors, including the presence of biofilm, sugar consumption, and the continuous processes of demineralization and remineralization in hard tooth tissue [2]. Dental decay affects both primary and permanent teeth, regardless of age. The initial process involves enamel demineralization, followed by pulp weakening, ultimately resulting in the loss of crown structure. The balance of protective and pathological factors influences the development and progression of caries [3].

Recurrent caries is the most common reason for replacing restorations of all kinds in general dental practice. Prevention of recurrent lesions by using fluoride-releasing restorative materials has been successful. Because recurrent carious lesions are localized and limited, alternative treatments to restorations such as crowns have been proposed, but this is not practical in all cases [4]. It is not advised to intentionally lose tooth structure. Failure to establish a complete seal in the restored tooth enables the persistence of cariogenic bacteria, leading to the recurrence of caries and ultimately resulting in restoration failure [5]. In the case of a minimally interventional approach (ART) to treat caries, recurrent caries have been reported more as cariogenic bacteria get trapped beneath GIC restorations due to manual removal of demineralized tooth tissue using hand instruments. Studies have shown that ART restoration rarely fails after six years due to the development of secondary caries. Hence, an effective way to tackle this challenge is by improving restorative materials that can offer strong hermetic seals and exhibit superior antimicrobial capabilities. Consequently, combining antibacterial agents with GIC might provide a therapeutic benefit. Nevertheless, it's important to note that the inclusion of antimicrobial agents in restorative materials often has an adverse impact on the physical and chemical properties of these materials [6]. Thus, assessing the compressive strength alongside the antibacterial effects of modified GIC is of significant importance.

There have been various reported endeavours to enhance the antibacterial capabilities of GICs by combining them with chlorhexidine hydrochloride, cetylpyridinium chloride, cetrimide, and benzalkonium chloride [7]. According to the literature, only chlorhexidine was widely incorporated into GIC, with all studies showing increased antibacterial activity in vitro [8], but it was not marketed due to chemical agents that can act on the cells of the pulp or the gingiva; hence, this study aimed to modify it with the bioactive component. The use of natural and herbal materials in dentistry is growing in popularity. The *Salvadora persica* tree, which is frequently used to make miswaks, is one of the most widely utilized materials. East Asia and West Africa are major distribution areas for *S. persica*. The term "Miswak" refers to a group of wooden sticks originating from diverse plant sources. Extracts from some of these plants have been utilized in the production of mouthwash and toothpaste [9]. *S. persica *extract demonstrated substantial antibacterial effectiveness against various oral pathogens, which encompassed *Candida albicans*, *Streptococcus mutans*, *Aggregatibacter actinomycetemcomitans*, *Lactobacillus acidophilus*, *Actinomyces naeslundii*, and *Porphyromonas gingivalis* [10]. In the realm of oral health, *S. persica* is known for its antibacterial, antifungal, anticariogenic, and antiplaque properties [11,12]. Miswak contains fluoride, phosphate, calcium and similar minerals found in the hydroxyapatite crystal of teeth [13]. This suggests its potential to aid in dental caries restoration; hence, this study was designed to modify GIC with Miswak extract and to assess both the antibacterial effectiveness and physical characteristics of Miswak-modified GIC.

## Materials and methods

Extract preparation

Miswak sticks were procured from Annai Aravindh Herbals Pvt Ltd., Chennai, India. After undergoing a five-day drying process, the Miswak sticks were utilized to prepare a mixture in a beaker, combining 100 mL of distilled water with 6 g of finely chopped Miswak sticks. This mixture was heated using a heating mantle, bringing it to a boil and maintaining a temperature between 60 and 70 degrees Celsius for a duration of 15 minutes. After filtration through Whatman No. 1 filter paper (Whatman Plc, Maidstone, UK), 80 ml of filtrate was collected in a separate Erlenmeyer flask, and this filtered extract was subsequently concentrated to a volume of 5 ml.

Specimen preparation

Type II GIC (GC Corporation) was utilized in this research study. Groups I, II, and III represent Miswak-modified GIC groups. In these groups, Miswak-modified GIC specimens were created in three different concentration ratios characterized by a powder GIC: Extract: Liquid GIC with Group I (2:1:1), Group II (3:1:2) and Group III (3:2:1). Group IV serves as the control group without any modification, employing conventional GIC. First, the conventional GIC components were mixed, and then the plant extract was added as per the groups. The resulting cement was placed in 6 mm-diameter, 2 mm thick cylindrical moulds, and samples were transferred to cylindrical wells. After application, disc-shaped samples were removed, measured, and recorded. Each group comprised 12 samples, six for *S. mutans* testing and the remaining six for *Lactobacillus*. Bacterial resistance was evaluated against *S. mutans* and *Lactobacillus *strains. Compressive strength, as per ISO 9917-1:2007 (4.0 mm diameter, 6.0 mm height), cylindrical moulds were prepared with 12 samples in each group. After an hour, samples were soaked in deionized water for 24 hours before testing.

Preparation of bacterial species and inoculation

*S. mutans* and *L. acidophilus* strains were acquired from the Department of Microbiology. After obtaining pure cultures, facultative strains were cultivated on Mueller-Hinton Agar (MHA), then subculture was separately introduced into tubes with 5 mL of sterile MHA broth. These tubes were incubated at 37°C for 24 hours, and the resulting suspension was subsequently adjusted to a 0.5 McFarland standard.

Minimal inhibitory concentration (MIC) assay

**Figure 1:**
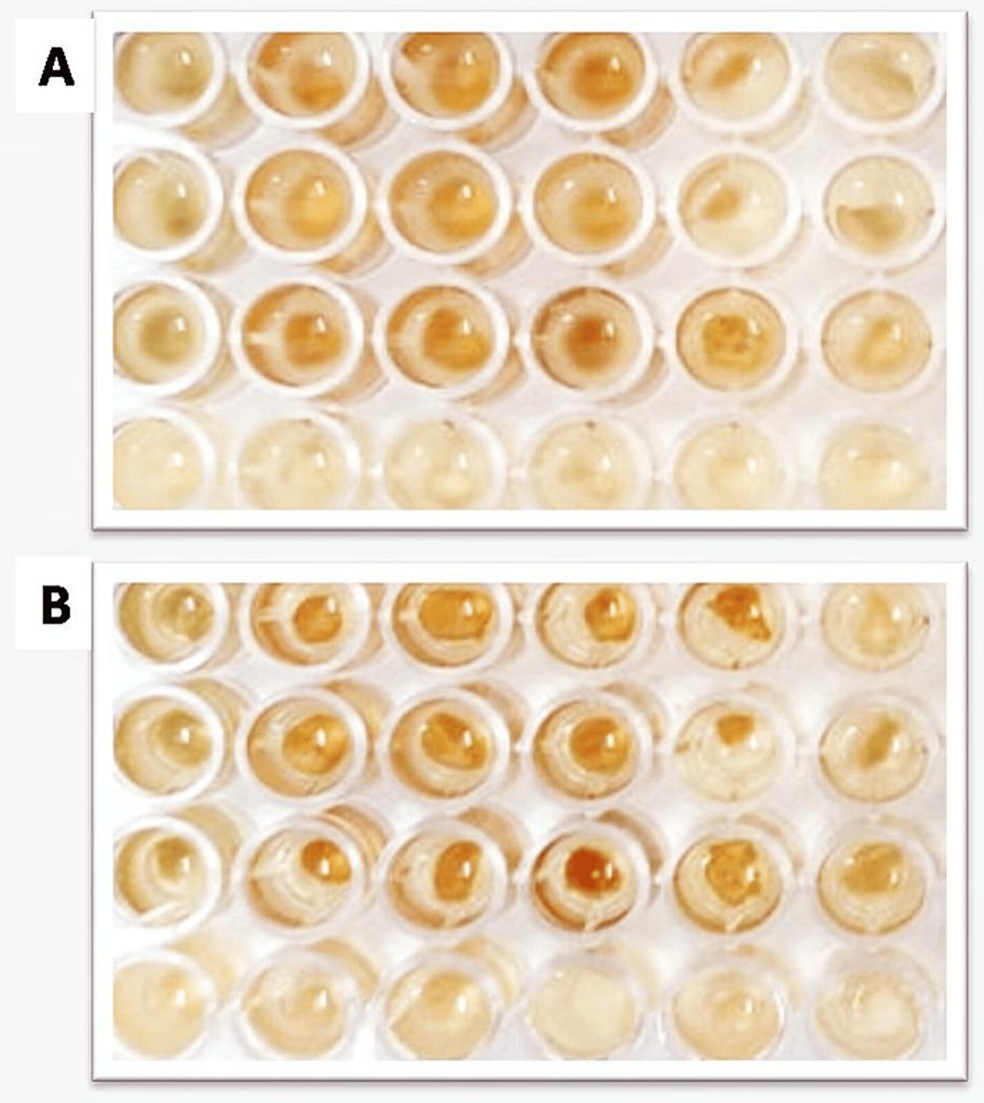
MIC assay in ELISA 96-well plate A - *Streptococcus mutans*, B - *Lactobacillus*

To evaluate the antimicrobial effectiveness of both modified and unmodified GIC, a total of 48 specimens, involving 12 samples per group, were used, with each well containing 200 µl of sterilized MHA broth. Then, 50 µL of bacterial suspensions at a concentration of 5 x 10^5 CFU/ml were added to the 24 wells (*S. mutans*) and the remaining for *L. acidophilus*. As per the groups, modified specimens containing different GIC concentrations (2:1:1), (3:1:2), and (3:2:1) and unmodified specimens were added (Figure 1).

Incubation was carried out under suitable conditions for varying time intervals (1h, 2h, 3h, 4h). The percentage of deceased cells was measured at 540 nm using an ELISA reader during specific time intervals.

Assessment of compressive strength

The diameter of each sample was assessed using a digital micrometre before placing them vertically in a universal testing machine (Instron, ElectroPuls®, Bangalore, IND). At a crosshead speed of 0.5 mm/min, a compression load was applied to the specimen's long axes until fracture, and readings were recorded.

Statistical evaluation

The data were input into Microsoft Excel (Microsoft® Corp., Redmond, WA) and analyzed using the Statistical Package for the Social Sciences (IBM SPSS Statistics for Windows, IBM Corp., Version 24.0, Armonk, NY) software. Descriptive analysis and repeated measures of analysis of variance (ANOVA) were performed to calculate the mean MIC values, comparing all the groups based on several time intervals. Tukey's post hoc test was assessed for pairwise comparison between groups. Compressive strength was assessed with repeated measures ANOVA to compare the groups, followed by Tukey's post hoc test (P < 0.05, 95% confidence) for pairwise comparison.

## Results

Antimicrobial efficacy against *S. mutans*

**Figure 2:**
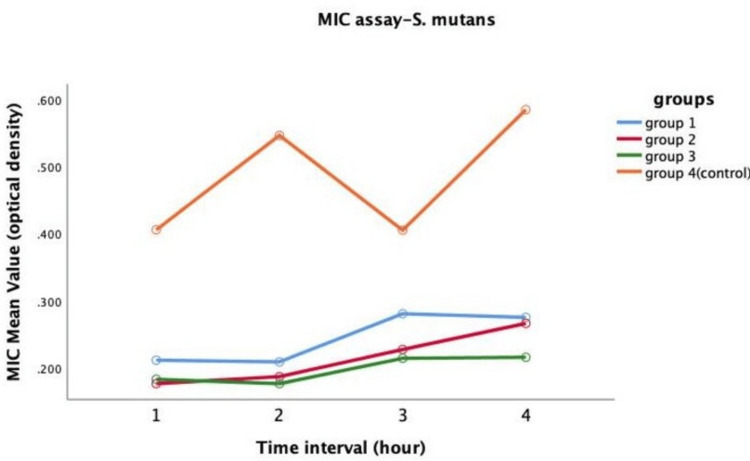
Antimicrobial efficacy on Streptococcus mutans between four groups MIC: minimal inhibitory concentration

Repeated measures of ANOVA tested modified and unmodified GIC's antibacterial activity against *S. mutans*. Group I, Group II, and Group III exhibited superior performance and statistically significant results compared to Group IV (control) (Figure 2). Also, Tukey's honestly significant difference (HSD) pairwise comparison test showed a significant difference between Group IV and the other three groups (Table 1).

**Table 1:** Pairwise comparison of antimicrobial efficacy on Streptococcus mutans between four groups *P value was significant at 0.05, P value was derived from multiple comparisons of Tukey's honestly significant difference (HSD) test.

Pairwise comparison	Mean difference	95% CI		P-value
		Lower	Upper	
Group I vs Group II	0.2983	0.027	0.032	0.001*
Group I vs Group III	0.046	0.044	0.049	0.001*
Group I vs Group IV	0.241	0.239	0.244	0.001*
Group II vs Group III	0.169	0.014	0.019	0.001*
Group II vs Group IV	0.271	0.269	0.273	0.001*
Group III vs Group IV	0.288	0.286	0.290	0.001*

Antimicrobial efficacy against *Lactobacillus*

**Figure 3:**
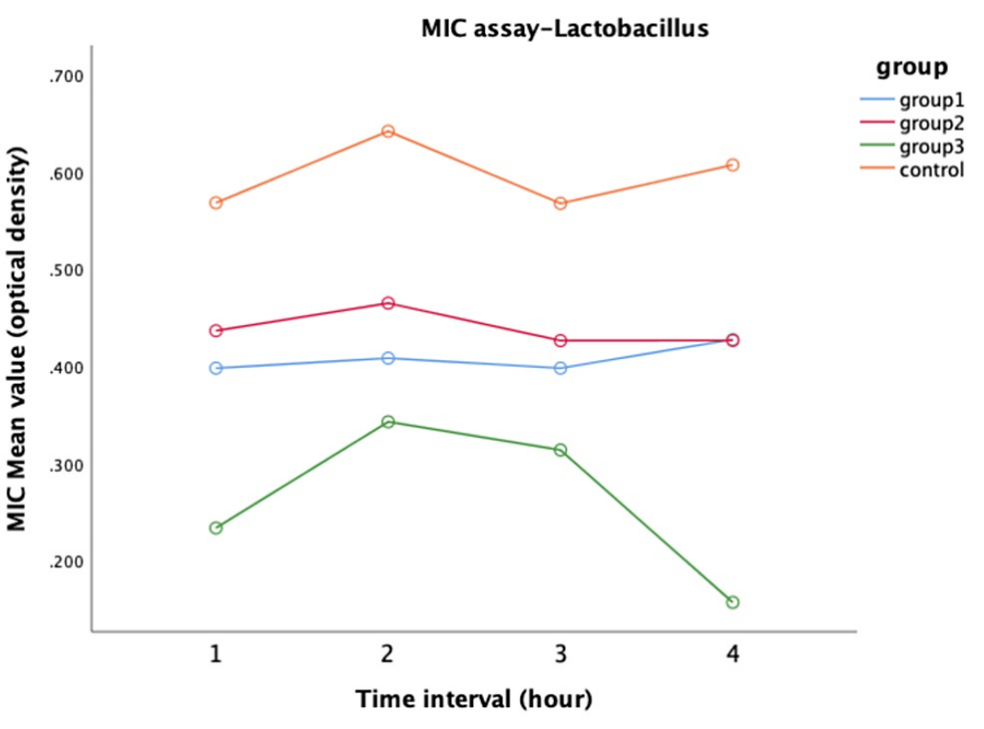
Antimicrobial efficacy on Lactobacillus between four groups MIC: minimal inhibitory concentration

For *Lactobacillus*, a repeated measure of ANOVA demonstrated that the modified groups exhibited notable antimicrobial activity, displaying superior performance compared to the control group (Figure 3). Pairwise comparisons for *Lactobacillus *revealed a significant difference (p<0.05) between Group IV and the other groups (Table 2), highlighting the pronounced antibacterial activity of Miswak-modified GIC over conventional GIC.

**Table 2:** Pairwise comparison of antimicrobial efficacy on Lactobacillus between four groups *P value was significant at 0.05, P value was derived from multiple comparisons of Tukey's honestly significant difference (HSD) test.

Pairwise comparison	Mean difference	95% Confidence interval		P-value
		Lower	Upper	
Group I vs Group II	0.030	0.029	0.031	0.001*
Group I vs Group III	0.146	0.144	0.147	0.001*
Group I vs Group IV	0.188	0.186	0.189	0.001*
Group II vs Group III	0.176	0.175	0.177	0.001*
Group II vs Group IV	0.157	0.156	0.158	0.001*
Group III vs Group IV	0.334	0.332	0.335	0.001*

Compressive strength

The compression load was applied to the specimens, and values were recorded. A one-way ANOVA showed a statistically significant difference between all groups with a p-value of 0.001 (Table 3). The pairwise comparison revealed Group II and Group III differed significantly from Group IV, with Group IV (control) showing better performance in compressive strength; however, there was no significant difference between Group I and Group IV (control), indicating similar performance of these groups with a p value of 0.995 (p > 0.05) (Table 4).

**Table 3:** Comparison between groups for evaluation of compressive strength *Significant at 0.05, P value was derived by one-way analysis of variance (ANOVA).

Group	n	Mean ± SD	95% CI		P value
			Lower	Upper	
Group I	12	62.51±5.54	58.99	66.03	0.001*
Group II	12	41.49±3.6	39.20	43.78	
Group III	12	15.23±4.96	12.07	18.38	
Group IV	12	62.69±2.58	60.44	63.73	

**Table 4:** Pairwise comparison for evaluation of compressive strength *significant difference at p<0.05, P value was derived from Tukey Post hoc test.

Pairwise comparison	Mean difference	95% CI		P-value
		Lower	Upper	
Group I vs Group II	21.025	16.30	25.74	0.001*
Group I vs Group III	47.284	42.56	52.00	0.001*
Group I vs Group IV	0.425	4.29	5.14	0.995
Group II vs Group III	26.258	21.53	30.97	0.001*
Group II vs Group IV	20.600	15.87	25.32	0.001*
Group III vs Group IV	46.85	42.13	51.57	0.001*

## Discussion

Although dental caries treatments do not always completely eliminate all microorganisms, some remain in residual tissue. When restorations lack effective hermetic seals, the continued presence of cariogenic bacteria can result in recurrent caries and, consequently, the failure of the restoration [14]. To address this challenge, one potential solution is the adoption of dental materials that exhibit properties capable of inhibiting and eradicating bacteria, both bacteriostatic and bactericidal in nature. Introduced by Wilson and Kent in 1972, conventional GICs are tooth-coloured, chemically bonded materials that are inherently anti-caries and contain fluoride, a widely used compound in dentistry. It has a therapeutic effect through its release properties. The ability of GICs to deliver fluoride continuously over an extended period of time offers anti-caries potential that indicates a reduction in caries adjacent to the restoration [15], but again, it cannot inhibit a broad spectrum of microorganisms. Also, cavities treated with ART (atraumatic restorative treatment) may retain infected dentin; thus, GIC proves ineffective in halting the progression of caries, resulting in restoration failure; hence, improving restorative materials to enhance the physical and antibacterial attributes of conventional GIC is necessary. Plant-based phytochemicals are gaining prominence as an alternative to commercial antimicrobial agents. They offer additional benefits, such as traditional medicinal use and cost-effectiveness, while avoiding issues of antibacterial resistance. One example is Miswak, an herbal antimicrobial agent extracted from the *S. persica* plant.

The current results of the study prove the superior antimicrobial effectiveness of Miswak-modified GIC against *S. mutans*. These findings are consistent with the work done by Sofrata et al. who reported on *S. persica*'s antibacterial properties against oral microorganisms, including *S. mutans* and *L. acidophilus* [16]. An in-vivo study by Kabil NS et al. revealed that the incorporation of Miswak into GIC led to superior antibacterial properties when compared to standard GIC, aligning with our current in vitro findings [17]. Furthermore, a prospective case-control observational study involving Miswak users and non-users revealed a significantly higher prevalence of dental caries among non-users. This study, conducted on 240 schoolchildren over two years, assessed the decayed-missing-filled (DMF) index. The lower incidence of dental caries among Miswak users suggests its potential role in caries prevention [18]. In another study by Lamia Singer et al., it was observed that blending plant extracts like *S. persica*, *Olea europaea*, and *Ficus carica* with GIC resulted in increased antimicrobial activity against *S. mutans* and *Micrococcus luteus*, particularly at higher concentrations [19]. The Miswak extract possesses a range of antimicrobial and antifungal attributes attributed to its content of chlorides, trimethylamine, fluoride, silica, saponins, sulfur, flavonoids, and phenols. Its b-sitosterol and m-anisic acid both contribute to its antibacterial activity [19]. A study conducted by El Tatari et al. showed that Salvadoran Persica Extract (SPE) combined with GIC showed promising results in antimicrobial tests, especially against *S. mutans* [20]. Kalpavriksha AJ et al. found that GIC with 1% CHX and Miswak extract was equally effective against *S. mutans* and *Streptococcus sobrinus* [21]. Ashouri et al. study showed that adding lyophilized Miswak to GIC improved its antimicrobial efficacy [22]. One of the studies investigated *S. persica* capacity to prevent collagen breakdown in demineralized dentin, where an aqueous *S. persica* extract was applied to demineralized bovine root dentin samples, which under a light microscope revealed that the extract shielded the dentin's collagen matrix from enzymatic degradation by collagenase. This proves a potential role for *S. persica* in caries prevention [23]. Apart from antimicrobial properties, this current study evaluated the compressive strength of Miswak-modified GIC.

The performance of a material in therapeutic contexts is dependent upon its capacity to bear pressure, stress, and strain. Compressive strength is the primary measure for characterizing dental cement. Hence, it was imperative to evaluate the compressive strength when modifying GIC. The compressive strength test took place after a 24-hour storage period, providing an optimal timeframe for testing its mechanical properties. A study by El Tatar showed that SPE combined with GIC at a concentration of 1% SPE maintained the same level of compressive strength as that of the control group [20], and this is consistent with the present study, where a lower concentration of the extract, i.e., Group I (2:1:1), unaltered the compressive strength. Farret et al. [24], Pavithra et al. [25], and Marti et al. [26] also noted similar results, indicating that the addition of antimicrobial agents at particular concentrations had no impact on the compressive strength characteristics of GIC.

Hence, the clinical significance of GIC infused with Miswak extract is noteworthy, primarily due to its potential effectiveness in combating *S. mutans* and *Lactobacillus*, pivotal contributors to caries development, including secondary caries. By incorporating this bacteriostatic agent, it becomes possible to impede caries progression and mitigate the risk of restoration failure. This approach could prove valuable in the treatment of patients with deep caries, early childhood caries, rampant caries, and high caries susceptibility. A limitation of the present study is its omission of intraoral variables such as masticatory stress, moisture levels, and potential discrepancies by operators. Further extensive research is required in regard to the bonding effects of GIC material's long-term stability and performance, which can promote a novel natural bioactive restorative material.

## Conclusions

The findings of the present study suggested that a lower concentration of Miswak extract has the potential to enhance antimicrobial properties without compromising the material's strength. Therefore, Miswak containing GIC can be a promising restorative material. Further studies are recommended to investigate setting time, fluoride release, and in vivo behaviour.
